# Exception to the Puppe’s rule reloaded and a warning in the interpretation of skull fractures: they run even through recent craniotomy burr holes

**DOI:** 10.1007/s12024-024-00829-0

**Published:** 2024-05-11

**Authors:** Alberto Amadasi, Lorenzo Franceschetti, Larissa Amadasi, Lars Oesterhelweg

**Affiliations:** 1 Institute of Legal Medicine and Forensic Sciences, University Medical Centre Charité, University of Berlin, Turmstr. 21, Building N, 10559, Berlin, Germany; 2 Department of Biomedical Sciences for Health, Section of Forensic Medicine, University of Milan, Milan, Italy

**Keywords:** Forensic anthropology, Cranial fractures, Puppe’s rule, Craniotomy burr holes, Exception

## Abstract

A possible “exception” to Puppe’s rule regarding the intersection of skull fractures has been previously addressed due to the observation that skull fractures can pass through old and remodeled craniotomies. In a further case presented herein, however, it was shown that cranial fractures are also able to pass through recent burr holes, a phenomenon never previously described. A 63-year-old man sustained a self-inflicted gunshot wound to the right temple region, with an exit wound in the left temporal region. Twenty-five days prior, the patient had undergone parietal craniotomy for the evacuation of a subdural hematoma secondary to glioblastoma. Among the fracture lines originating from the exit wound, one traversed the craniotomy hole, terminating approximately 1.4 cm beyond its contralateral margin. This illustrates that cranial fractures possess the capability to cross “fresh” burr holes that have not undergone to bone remodeling. Consequently, the evaluation of Puppe’s rule should be reconsidered, particularly in cases of gunshot injuries, wherein fractures pass through full-thickness circular lesions (such as entry and exit wounds). The varied scenarios underscore the potential for fractures to “pass through” these burr holes if they have not themselves generated fracture lines, as may be the case with entry holes with circular lesions without fractures.

## Introduction

Puppe’s rule, named after the forensic pathologist Puppe, who enunciated it more than 100 years ago, stands as one of the cornerstones in the interpretation of the development of skull fractures and thus of the sequence of injuries. Puppe’s rule posits that in cases of traumas produced at two different times, subsequent fractures cannot surpass pre-existing fractures and thus that the fractures resulting from a later trauma will interrupt by fractures that were already caused by the earlier traumatic event [1–6]. This theory has also been successfully applied to gunshot wound analysis, particularly in discerning between entry and exit wounds [7, 8]. Previous studies have demonstrated that gunshot wounds and associated beveling are interrupted and delimited by pre-existing fracture lines. Moreover, fractures from exit gunshot wounds stop at the edge of the entry wound [9–12]. In a recent article [13], a particular “exception” to Puppe’s rule was observed, namely that fractures in the presence of old and remodeled craniotomy holes are able to “pass through” them without stopping at the margins of the hole, hypothesizing a role of bone remodeling on energy transmission.

The present case presents a novel finding: fracture lines pass through “fresh” craniotomy holes, occurring in this instance 25 days postoperatively. Such an occurrence has not been described in the forensic literature and prompts a reevaluation of the relationship between fracture lines and cranial burr holes.

## Case report

A 63-year-old man was discovered deceased in his residence by the wife after a self-inflicted gunshot wound to the head. A semi-automatic ‘Glock’ pistol, lacking a bullet in its magazine, was recovered near the body, which was subsequently found on the room’s floor. The caliber of the full-metal-jacket bullet was 9 × 21 mm, with no discernible alterations. The decedent’s wife revealed his medical history, noting a diagnosis of glioblastoma and recent craniotomy surgery performed 25 days prior. The craniotomy aimed to relieve intracranial hypertension caused by the tumor and a subdural hematoma, as well as to conduct an extensive biopsy of the neoplasm. No surgical complications were reported, with the procedure executed without affecting the surrounding bone. The decedent’s deteriorating health condition had precipitated depressive symptoms and suicidal ideation.

**Fig. 1:**
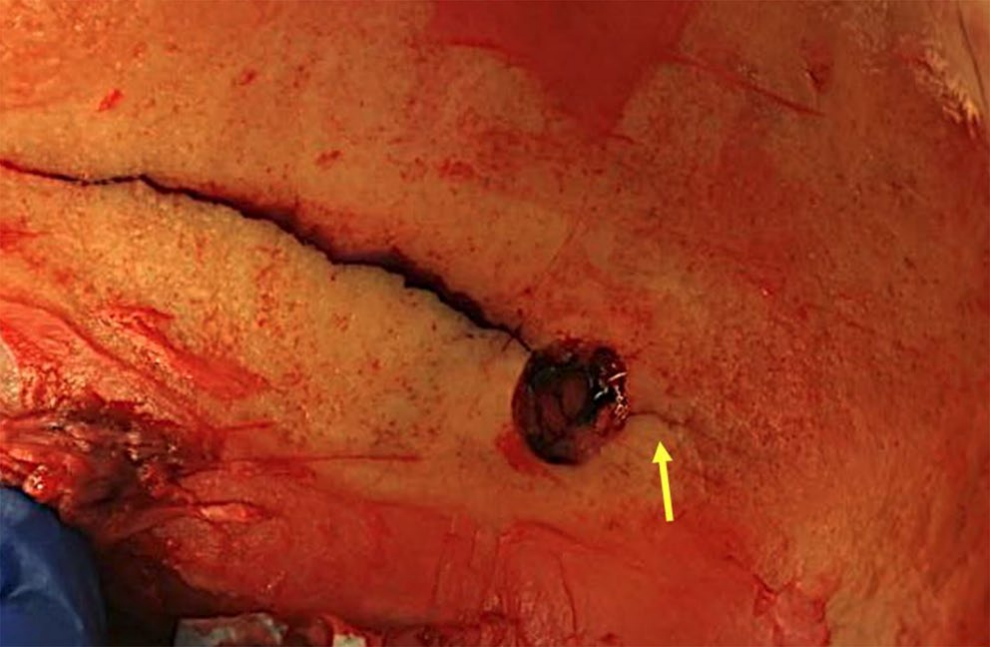
Fracture line on the cranial vault at autopsy with extension of the fracture beyond the craniotomy hole

**Fig. 2:**
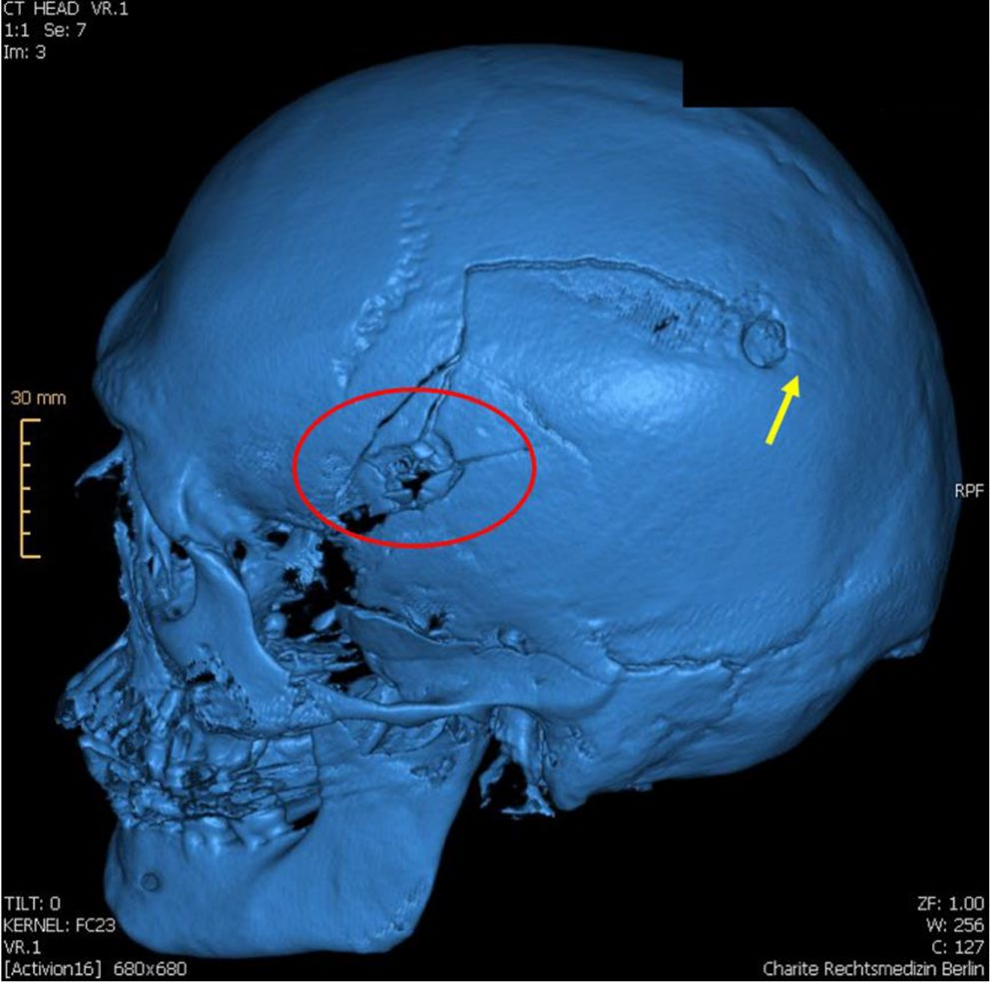
3d reconstruction of the skull with fracture line starting from gunshot exit wound (red circle). Extension of the fracture indicated with yellow arrow

**Fig. 3:**
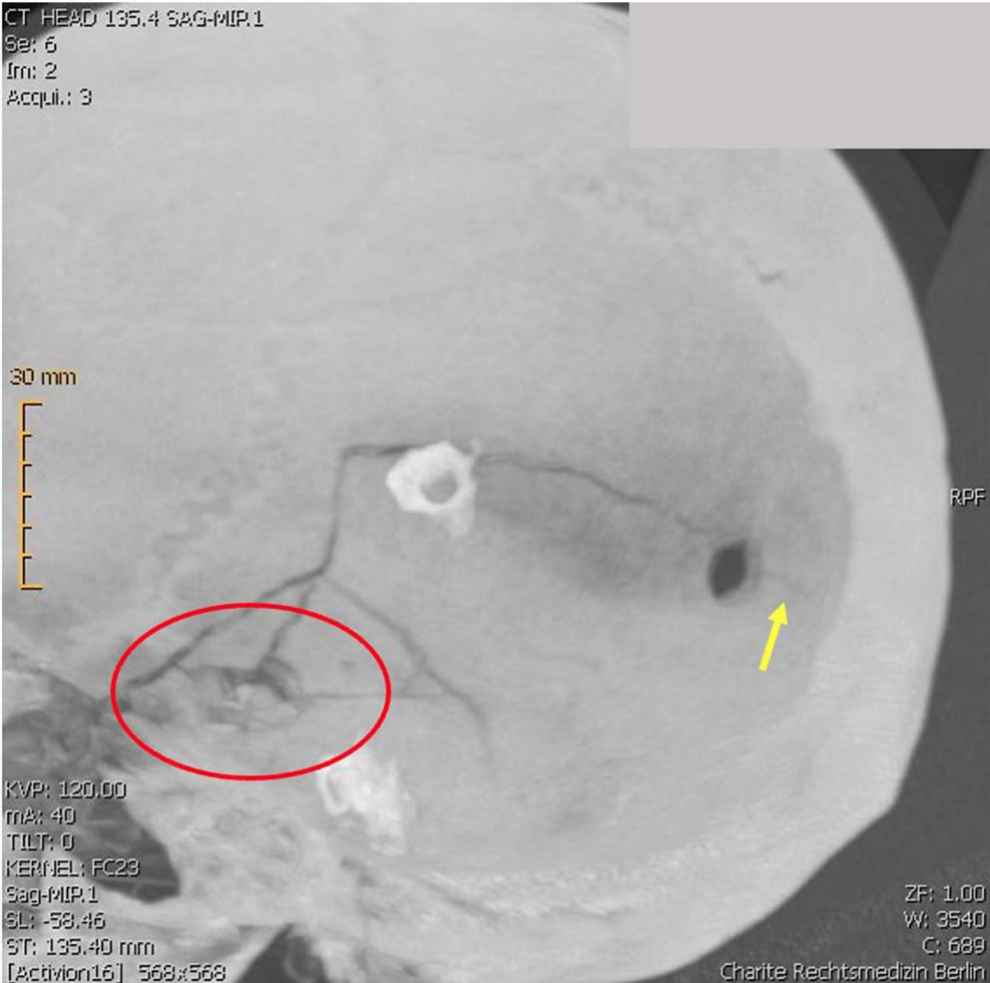
MPR-Image of the skull with fracture line starting from gunshot exit wound (red circle). Extension of the fracture indicated with yellow arrow

Prior to the autopsy, the body was subjected to a CT scan with 3D reconstruction. At the autopsy, an entrance wound on the right temple and an exit wound on the left temple were found. The entry wound showed a round shape, regular margins and blackening of both skin and bone margins, indicative of a close-range firing. Internal beveling and several fracture lines were evident in the skull. The exit wound on the left temple showed a larger diameter, irregular skin margins, external beveling, and bone fragments embedded in the skin. Additionally, a drill hole with regular margins, approximately 0.8 cm in diameter, was observed in the left parietal region, positioned 9.2 cm from the central point of the exit wound. Notably, a fracture line originating from the upper margin of the exit wound extended superiorly, nearly vertically, before curving posteriorly toward the anterior margin of the drill hole, and subsequently continued along the opposite margin for approximately 1.4 cm (Figs. 1, 2 and 3). The appearance of the fracture was of a complete fracture (and therefore not a fissure) observable on both the outer and inner sides of the calvarium.

## Discussion

The examination of cranial fractures constitutes a wide-ranging and multifaceted topic within the disciplines of forensic pathology and anthropology. The correct assessment of fracture lines in the skull could be crucial to assess causes and manners of death. Furthermore, discerning the temporal sequence of multiple traumatic events that result in fracturing of a solitary cranium poses an essential yet convoluted endeavor [14–18]. In this context, macroscopic analysis may rely on the Puppe’s rule, as outlined previously. On the basis of a sort of exception to the presumed irrefutability of Puppe’s rule on skull fractures [1–7], it was shown that in an ‘unexpected’ way, skull fractures were able to pass through existing holes such as holes from previous craniotomies, the margins of which are thus largely remodeled [13]. This phenomenon was attributed to the transmission of forces facilitated by bone remodeling, resulting in fracture deformation. As a result of the previous study and on the basis of the existing literature, it was postulated that the existence of a recent burr hole with non-remodeled edges would disrupt fracture line continuity. Until then, it had never been discussed how a skull fracture behaves when it encounters a craniotomy hole. Instead, the present case provides a significant observation: cranial fractures can indeed pass through recent craniotomy burr holes, with energy transmission deforming the hole and continuing along the margins to the opposite side. This aspect of the behaviour of skull fractures in the presence of craniotomy holes has therefore only been described once before, but it is very important to emphasise that this can be observed with both recent and old burr holes and is a fundamental concept of differential diagnosis, especially when the patient’s clinical history and previous performance of possible neurosurgical operations are unknown. All this leads to the hypothesis that the specific behavior of the fracture is due to the integrity of the hole and not to its remodeling. Consequently, fractures originating from the exit hole, or from a second impact in the case of blunt force, would be interrupted at the margins of the initial entry hole (or the hole given by the first impact), since the margins of the latter are no longer “intact”, in the sense of no longer being in 360-degree continuity due to the prior development of fractures. Nevertheless, it is important to note that in cases involving penetrating injuries from small-caliber projectiles or sharp objects, the occurrence of a bone defect (hole) does not result in any additional fracture [16, 19–21]. In such instances, therefore, a craniotomy hole, given the regularity of its margins, can macroscopically resemble a gunshot entry wound (for example, a gunshot entry wound from small-calibre bullet).

In these cases, it must therefore be taken into account that fractures originating from a second impact could cross the hole of the first impact and thus resemble entrance hole fractures. In fact, looking at the fracture lines shown in the figures and assuming that that is an entry gunshot wound, one might mistakenly think that those fractures were caused by the effects of a direct impact.

This should definitely be pointed out and taken into account: a differential diagnosis between fractures starting at the site of trauma or fractures reaching it from a secondary site of trauma. Such a crucial issue has never been described in the literature, but it is something that must be taken into account in the complex evaluation of the origin and course of skull fractures when assessing the sequence of the injuries.

## Key points

1. Puppe’s rule is a fundamental principle in the evaluation of skull fracture sequences and has never been practically refuted.
2. It has been shown in some cases that fractures can pass through the holes of previous craniotomies, both recent and old, thus not stopping at the edge of the hole.
3. These characteristics are crucial and must be taken into account in the differential diagnosis of the origin of skull fractures.

## Data Availability

The Authors confirm that the data supporting the findings of this study are available within the article.
